# Large-Scale Marsh Loss Reconstructed from Satellite Data in the Small Sanjiang Plain since 1965: Process, Pattern and Driving Force

**DOI:** 10.3390/s20041036

**Published:** 2020-02-14

**Authors:** Fengqin Yan

**Affiliations:** State Key Laboratory of Resources and Environmental Information System, Institute of Geography and natural resources, Chinese Academy of Sciences, Beijing 100101, China; yanfq@lreis.ac.cn; Tel.: +86-010-6488-9676

**Keywords:** spatiotemporal pattern, marsh, Small Sanjiang Plain, land use, wetland restoration

## Abstract

Monitoring wetland dynamics and related land-use changes over long-time periods is essential to understanding wetland evolution and supporting knowledge-based conservation policies. Combining multi-source remote sensing images, this study identifies the dynamics of marshes, a core part of wetlands, in the Small Sanjiang Plain (SSP), from 1965 to 2015. The influence of human activities on marsh patterns is estimated quantitatively by the trajectory analysis method. The results indicate that the marsh area decreased drastically by 53.17% of the total SSP area during the study period, which covered the last five decades. The marsh mostly transformed to paddy field and dry farmland in the SSP from 1965 to 2015, indicating that agricultural encroachment was the dominant contributor to marsh degradation in the area. Analysis of the landscape indexes indicates that marsh fragmentation was aggravated during the past five decades in the SSP. Trajectory analysis also indicated that human activities have acted as the primary driving force of marsh changes in the SSP since 1965. This study provides scientific information to better understand the evolution of the wetland and to implement ecological conservation and sustainable management of the wetlands in the future.

## 1. Introduction

Wetlands, the kidneys of the landscape, play an important role in hydrological and carbon cycles, influencing groundwater recharge, gross water balance, flood response, greenhouse gas (GHG) emissions, carbon storage, and biodiversity maintenance [1,2,3,4]. Compared to other ecosystems of the same climate zone, the productivity of wetland ecosystems is usually much higher [5]. However, wetlands are quite sensitive to human disturbances, as well as climate change, and this leads to a severe degradation and loss of wetlands globally [6,7,8,9,10]. Wetland degradation or loss will bring about a series of ecological and environmental problems, including biodiversity loss, reduced ecosystem services, soil erosion, and increased flood risk [11,12,13,14]. Wetland protection and restoration are essential all over the world to promote sustainable development, especially in areas where wetlands have been damaged [13,14,15,16]. Clarifying the spatiotemporal patterns of wetlands and distinguishing the contributions of different factors to wetland changes are key issues for understanding wetland evolution and implementing wetland protection and restoration activities [17,18,19]. Therefore, future studies should quantify the influence of different relevant factors, such as human activities, on wetland areas and their distribution, to better understand wetland evolution.

Satellite images, an important source for obtaining historical land-use information, may also be the most inexpensive and effective option [20,21,22]. However, the unavailability of satellite data before 1972 has become a constraint for monitoring historical land-use patterns during this period. Usually, model reconstruction, e.g., using a Cellular Automata (CA) model to reconstruct historical land-use patterns, was the main method to obtain historical land-use maps before 1972 [23,24]. However, model reconstruction is accompanied by uncertainty to some extent. Satellite images can provide great convenience in detecting historical land-use patterns. The CORONA satellite, which was launched in 1958, can be used as a potential data source for environmental monitoring [25,26,27,28]. The CORONA satellite, during 1960–1972, took many images that can serve as an important dataset for detecting land-use changes during this period [28,29,30,31,32,33,34]. The CORONA satellite was not public before 1995, which is one of the reasons why these images were not widely used. Acquired with a telescopic camera system, the CORONA images are black-and-white at 7-micron (3600 dpi) or 14-micron (1800 dpi) resolution. The best product of the CORONA mission has a resolution of approximately 6 feet (1.8 m). Generally, classifying land-use patterns from panchromatic images with a high resolution, like the ones from CORONA, is challenging as a result of spectral limitations [26]. Some efforts have been made to acquire land-use maps using CORONA images, such as monitoring irrigation ponds, soil erosion, and cultivated land [22,25,26,35,36,37,38,39]. To cover the coarse spectral limitations of CORONA images, Gurjar et al. combined contrast, textural, and geometrical information to perform classification [26]. Chen et al. used textural features, instead of the spectral information, for supervised land-use classification of CORONA images. In this study, CORONA images were used to obtain land-use distribution information in 1965. In this study, textural features, shape, structure, and tone information, as well as ancillary data, were combined to perform the classification. CORONA images were re-sampled to 30 m to maintain consistency with the Landsat images. Integrating multi-source RS images is generally the most economically feasible way to acquire historical land-use maps in many cases. However, the lack of related historical land-use data creates a problem of accuracy validation. Extensive field surveys, historical records (such as aerial images and statistic data), and interviews with local residents and experts, as well as Unmanned Aerial Vehicle (UAV) images, were applied to validate the accuracy of this study.

Bi-temporal detection is the general method to analyze land-use changes [40,41,42] and is easy and convenient to carry out. Bi-temporal detection analyzes land-use changes based on a two-epoch timescale. However, some information is ignored by this bi-temporal detection method. Recovering the history of land-use changes and linking spatiotemporal changes to different driving forces (human activities and natural factors) is usually necessary to better understand the land-use evolution process. Trajectory analysis is a method used to study and discover the trends of land use and land cover changes (LULCC) in a time series. The trajectory analysis method, which can recover land-use history as well as link land-use changes to different driving forces, has been widely used to quantitatively study the influence of human activities on land-use changes [18,43,44,45]. Additionally, trajectory analysis can be applied to illustrate the trends of land-use patterns over time [44,45]. The trajectory analysis method has also been applied to estimate future land-use changes over time [43,44,45,46]. In this study, trajectory analysis was adopted to better understand the marsh evolution process and to quantitatively research the influence of human factors on marsh loss.

As one of the core parts of wetlands, marsh is defined as an area that is frequently or continually inundated with soft-stemmed vegetation that is adapted to a saturated soil. The Sanjiang Plain contains the largest freshwater marsh in China. This study focuses on the dynamic changes of marsh in the hinterland of Sanjiang Plain, the Small Sanjiang Plain (SSP). Since the 1950s, the marshland in the SSP has experienced large-scale reclamation [47,48,49]. Given the importance and necessity of wetland protection and restoration, it is necessary to link marsh transformations to different land-use changes and various influencing factors. Based on multi-source remote sensing (RS) data containing CORONA, Landsat TM (Thematic Mapper), and OLI (Operational Land Imager) images, this study describes marsh dynamics as well as its related land-use changes. Additionally, we traced marsh transformation for every location and quantitatively distinguished the effects of different factors on marsh evolution. In particular, the objectives were (1) to illustrate the marsh patterns and related land-use changes in the SSP during 1965–2015; (2) to analyze the landscape patterns of marshes in the past five decades through landscape indexes; and (3) to trace the marsh change paths and quantify the influence of human disturbances on marsh loss in the SSP.

## 2. Materials and Methods

### 2.1. Study Area

**Figure 1:**
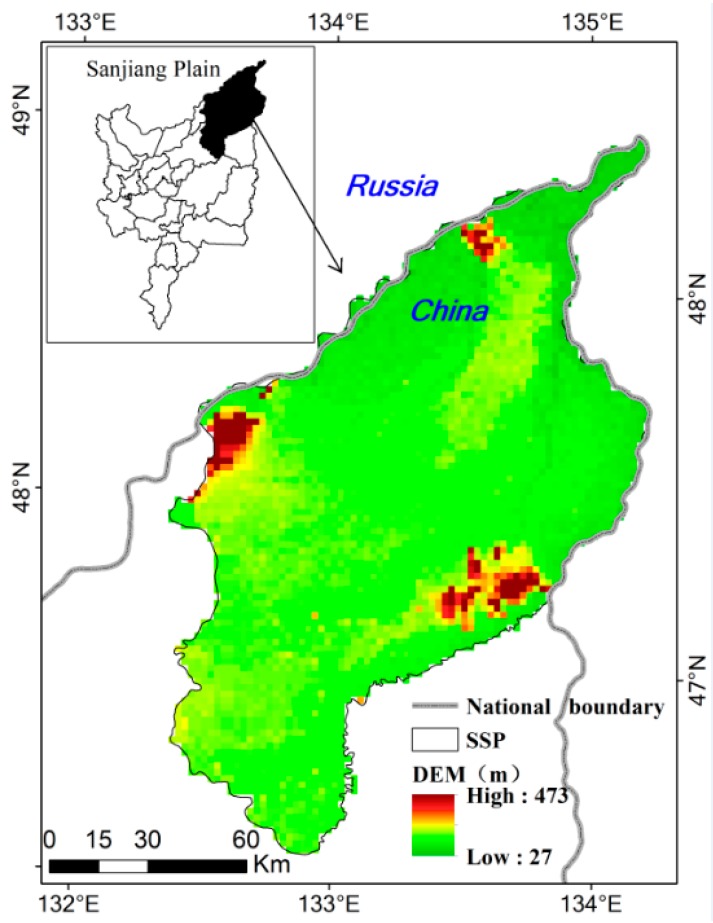
The study area. SSP, Small Sanjiang Plain.

The Small Sanjiang Plain (SSP) (46°50′05″–48°27′56″ N, 130°32′57″–135°05′26′′ E) is a floodplain in Northeast China, formed by the alluviation of three rivers (Amur, Ussuri, and Songhua) (Figure 1). Several important National Nature Reserves, including Sanjiang National Nature Reserve, Honghe National Nature Reserve, and Bachadao National Nature Reserve, are located in the SSP. The total area of the SSP is approximately 1.6 million ha, in which four counties are included. The yearly mean temperature in the SSP ranges from 1.4 to 4.3 °C with July and January being the warmest and coldest months, respectively. The yearly mean precipitation in the SSP ranges from 500 to 650 mm. The SSP has been suffering from both climate warming and extensive human disturbances since the mid-1950s [18,48]. Large-scale natural wetlands have been reclaimed as cultivated land since the mid-1950s. Since the 1990s, the great value of wetlands has been recognized, and the government began to consider the importance of the protection of wetlands in this region. Therefore, wetland reclamation has decreased significantly. Large-scale conversion of paddy fields to dry farmlands began in the 1990s. The transformations from wetland to dry farmland and then to paddy fields were the main land-use change type in the SSP. Therefore, this study chose 1965, 1995, and 2015 to reveal the major land-use change process.

### 2.2. Data Source and Handling

**Figure 2:**
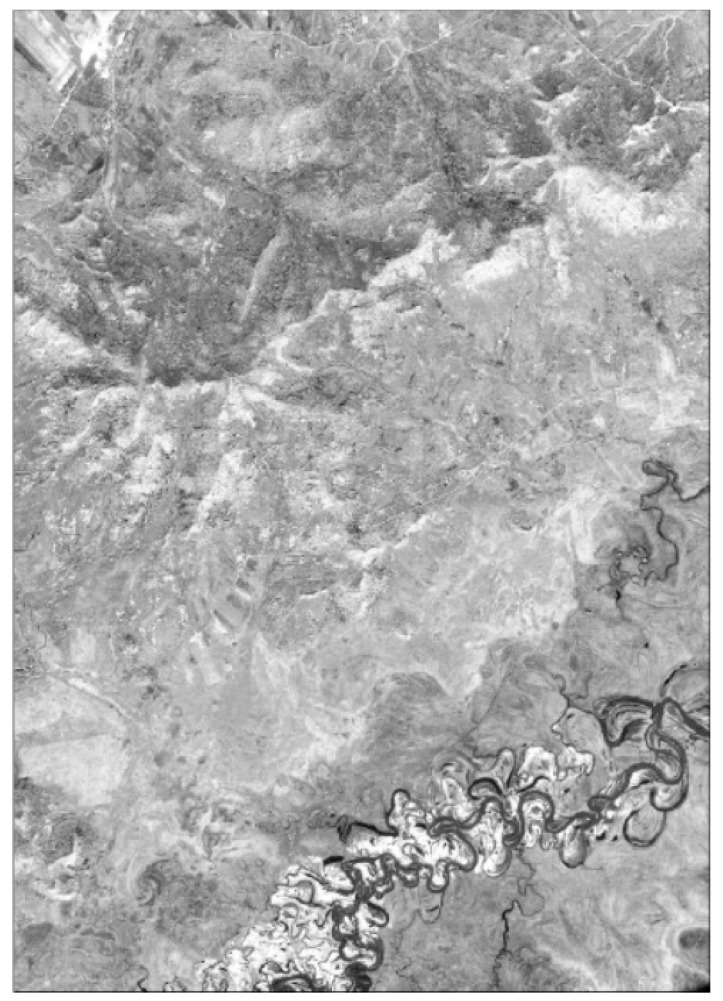
CORONA black-and-white images.

CORONA images, Landsat TM, and Landsat OLI are the main data sources used in this study to obtain land-use patterns during 1965–2015. In this study, 18 CORONA black-and-white images with a ground coverage of 14 × 188 (in kms) were used to map the land-use distribution patterns of 1965 (Figure 2). The CORONA images were acquired by a telescopic camera system and scanned as digital black-and-white images at a 7-micron (3600 dpi) or 14-micron (1800 dpi) resolution. The CORONA images used in this study have a spatial resolution of 6 feet (1.8 m). Eight CORONA satellite images acquired on 13 July 1964 and ten images acquired on 24 September 1966 were downloaded from Earth Explorer, USGS, to cover the whole study area. The CORONA images were re-sampled to 30 m to maintain consistency with the Landsat images. The principle behind the selection of RS images in order to obtain land-use data is that the imaging date (season) should be consistent. The imaging date of RS images in this study ranges from June to September. Image interpretation was mainly based on images obtained in the same month, with images from other months used as a supplement in the uncovered area. Georeferencing of CORONA images was carried out using Ground Control Points (GCPs). For each image scene, at least twenty evenly distributed points served as GCPs. The selected GCPs in the SSP were generally river intersections or road crossovers. Before further visual interpretation, it was essential to evaluate the georeferencing accuracy. The root mean squared (RMS) error of geometric rectification was no larger than 1.5 pixels (45 m) in this study. The minimum mapping unit in this study was no less than 36 pixels (3.24 ha).

With only one broadband 8-bit (0–255) gray level, the supervised classification method cannot be directly used on the CORONA images. Therefore, RS images were visually interpretated and digitized by the ArcGIS software in this study to obtain land-use classification in the SSP. Uniform quality control and integration to check for land-use data in different years were conducted to guarantee the high-quality and consistency of the land-use maps. Before developing our land-use data, field surveys were widely performed to generate interpreting samples. The interpreters were first trained to recognize different land-use types from CORONA/Landsat images. Then, the interpreters made use of the ArcGIS software to identify different land-use types on the computer screen, according to their understanding of spectral reflectance, structure, shape, textural features, and other information of the images. Finally, the interpreters drew the boundaries of different land-use types and added their attributes to the polygons. After the interpretation tasks were completed, the interpreters first checked the results by themselves, and then an interpreter with decades of interpretation experience checked the results a second time. If possible, the same interpreter was responsible for the interpretation of a certain region. In this study, topographic, vegetation, DEM, roads and rivers, and soil maps in this area were used as auxiliary data for interpretation. The post-classification comparison method has the disadvantage of error amplification in the spatial analysis process. To reduce this error, the outlines of land-use types were delimited by comparing RS images between intervals. For example, the vector lines of land-use types in 2015 were drawn by comparison with Landsat TM images of 1995 and Landsat OLI images of 2015 based on land-use maps of 1995 to maintain consistency in this study. A more detailed description of the prepossessing and interpretation of Landsat images can be found in our previous publications [50,51,52,53,54,55,56].

Many field surveys, aerial photos, historical field survey records, Statistical Yearbooks, and interviews with locals were adopted to evaluate the accuracy of our results [54,55,56]. Photos taken by cameras during field surveys were used as the main data to validate the land-use data in this study. Considering that marshes are usually distributed in remote place where traffic is often not convenient, Unmanned Aerial Vehicles (UAVs) were applied to perform field surveys and accuracy verification in 2015. UAV images were mainly used as a supplement to correct and check the interpretation results. A battery-powered quadrocopter can fly for approximately 23 min. When the UAV flies at an attitude of 200 m, it can acquire photos with a resolution of 5–6 cm [18]. Images obtained from the UAV are a relatively new way to obtain high-resolution images for observing the Earth’s surface. These images taken by UAVs have been widely used in scientific research, including forest monitoring, detecting rangeland, and mapping gully erosion [57,58,59]. UAV images were only applied for the year 2015 and were used as a supplement for the classification of land-use in this study. The verification points were randomly chosen at a ratio of 10%. Visual interpretation is usually labor intensive, but it usually has a relatively high accuracy. The overall accuracy of the four classes (forest land, grassland, water body, and settlement) was no less than 94.3% and that of the four subclasses (paddy field, dry farmland, marsh, and other unused land) was no less than 91.2%. For example, the average accuracy of land-use for 1995 was 92.9% [50].

### 2.3. Data Analyses

#### 2.3.1. Loss Area and Rate

The annual loss area (ALA, ha/year) and loss rate (LR, %) was calculated by [18]:

(1) $$ALA=\;A_{t2}-\;A_{t1}/\left(t_{2}-t_{1}\right)\times 100\%$$

(2) $$LR=\;A_{t2}-\;A_{t1}/A_{t1}\times 100\%$$

where ALA means the annual loss area, while LR represents the loss rate between time t_1_ and time t_2_. At_1_ and At_2_ are the areas of marshy wetland at time t_1_ and time t_2_, respectively.

#### 2.3.2. Landscape Pattern

In our study, six metrics, including Mean area (AREA_MN), Landscape shape index (LSI), Number of patches (NP), Landscape division index (DIVISION), Splitting index (SPLIT), and Aggregation index (AI), were applied to illustrate the landscape pattern changes of the marshland in the SSP (as is shown in Table 1). Fragstats software (Fragstats, 4.2, Oregon State University: Corvallis, OR, USA) was used to calculate these six metrics. All eight land-use types were calculated, but we only analyzed the landscape changes of the marsh.

#### 2.3.3. Trajectory Analysis

The trajectory code was calculated as in Equation (3) to capture the trajectory changes of marsh changes:

(3) $$Y_{i}={\left(G1\right)}_{i}\times 10^{n-1}+\;{\left(G2\right)}_{i}\times 10^{n-2}+\ldots {\left(Gn\right)}_{i}\times 10^{n-n}$$

where Y*i* and *n* indicate the trajectory code and the number of time intervals, respectively and (*Gn*)*i* represents the code of different land-use change types in patch *i* at a time node. Different land-use types were represented by numbers 1 to 8 in this study (Table 2). For example, number 1 and 2 were used to represent paddy fields and dry farmland, respectively.

Additionally, land-use types were grouped to 3 classes based on trajectory code Yi: human-induced, natural-evolution, and unchanged types. This study also breaks the “natural evolution” category into two subcategories: “natural evolution leading to marsh gain” and “natural evolution leading to marsh loss”. Table 3 lists the definitions and examples of different land-use change types related to marsh changes. Land-use types were classified as human-induced types whenever a land-use change is human-induced. The unchanged type means that the land-use type remains the same during the study period (such as code “777”).

## 3. Results

### 3.1. Spatio-Temporal Changes

#### 3.1.1. Percentage Changes

**Figure 3:**
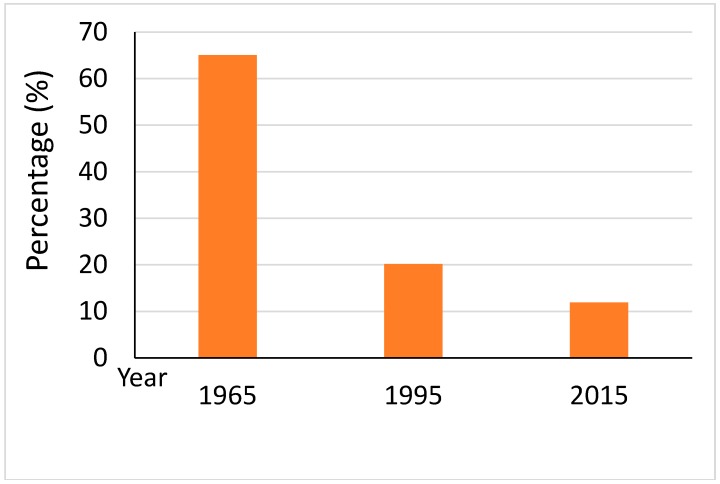
The percentage changes of the marsh area in the SSP since 1965.

**Figure 4:**
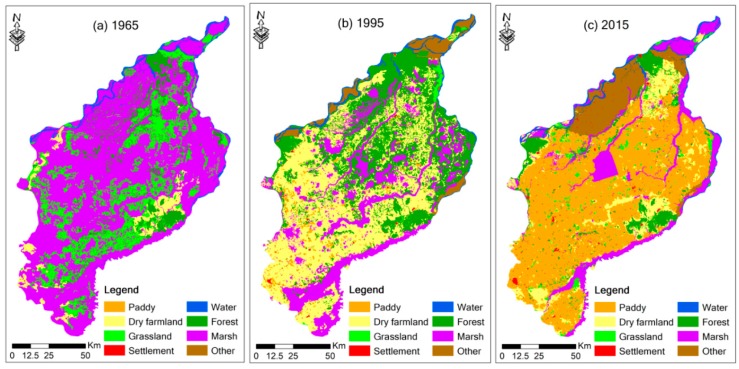
The spatial changes of land-use distribution in the SSP during 1965–2015.

Figure 3 demonstrates the area percentage of marsh in the SSP during 1965–2015. Statistical data indicate that the area percentage of marsh decreased from 65.07% in 1965 to 11.90% in 2015, with a drastic drop of 53.17% during the study period. The marsh area in the SSP declined drastically during period 1 (1965–1995) and then decreased slightly during period 2 (1995–2015). Figure 4 demonstrates the spatial changes of land-use in the SSP. It can be clearly seen from Figure 4 that the marsh was reclaimed as cultivated land on a large scale.

#### 3.1.2. Loss Area and Loss Rate

The loss area, annual loss area, and loss rate of marshes during different periods are shown in Table 4. Statistics indicate that marshes in the SSP decreased dramatically by 68.96% and 41.08%, respectively, during period 1 (1965–1995) and period 2 (1995–2015). The loss area during period 1 was 0.72 million ha, with an annual loss area of 23,982.89 ha/year in period 1. The loss area decreased to 0.14 million ha during period 2, with an annual loss area of 6652.36 ha/year in period 2. The dramatic decrease of marshland in period 1 is largely related to the “Food First” policy published in 1957 and the “Agricultural Modernization” policy implemented from 1978 to 1985. The “Food First” policy encouraged more people to participate in agricultural reclamation activities [18], while the “Agricultural Modernization” provided advanced equipment for large-scale reclamation and promoted large-scale transformation from marsh to cultivated land [48]. The loss rate of marshland showed a downward trend in period 2, mainly due to wetland protection policies and actions since the 1990s, such as the establishment of nature reserves.

### 3.2. Landscape Change

The landscape pattern characteristics are illustrated by the AREA_MN, NP, LSI, DIVISION, SPLIT, and AI indexes. LSI and AREA_MN (ha) show an obvious downward trend from 1965 to 2015, indicating a large-scale loss of marsh during the study period. The NP which represents the number of patches, decreased from 1383 in 1965 to 489 in 2015 (Table 5).

A smaller AI index represents less aggregation, while higher DIVISION and SPLIT indexes represent more fragments. This study indicated that the AI declined from 98.57 to 97.86, while the DIVISION grew from 0.68 to 1.00 during 1965–2015. In the SSP, the SPLIT rose from 3.09 to 696.43 during the last five decades. Changes in these three indexes show that the fragmentation of marshland was aggravated during 1965–2015 in the SSP. The changes during period 1 were more obvious than those during period 2.

### 3.3. Trajectory Computing

**Figure 5:**
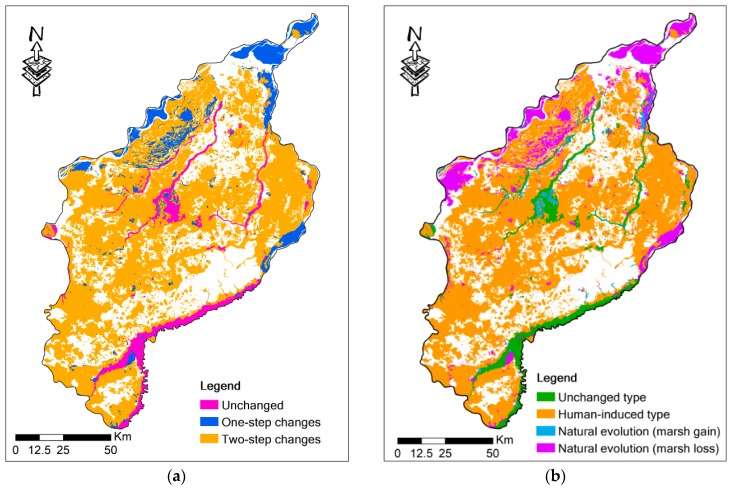
Trajectories of marsh changes, 1965–2015: (**a**) marsh changes with different steps; (**b**) marsh change types.

Figure 5 describes the trajectory changes of the marsh in the SSP. Figure 5a shows the marsh change with different steps. In the past five decades, unchanged types of marsh occupied only 7.60% of the SSP’s area, while the percentages of one-step and two-step changes were 28.15% and 64.25%, respectively. The above trajectories of marsh changes show the weak stability of the marshland in the SSP in the last five decades.

20 types were included in the one-step changes (Table S1 in Supplementary Materials), mainly contributed by the conversion from marsh to paddy and dry farmland. Statistic indicated that the percentage of one-step change was 28.15%, of which the conversion from marsh to cultivated land accounted for 20.09%. “771” (Marsh → Marsh → Paddy) accounted for the biggest proportion (10.89%) among one-step changes, followed by “772” (Marsh → Marsh → Dry farmland, 5.31%), and “733” (Marsh → Forest → Forest, 2.55%). The ratio of transformation from other land-use types to marsh made up merely 0.64%. All these changes showed that marsh in the SSP has been reclaimed intensely since 1965, and once they were destroyed, they would be hard to restore.

There were 59 types in the two-step change trajectories (Table S2 in Supplementary Materials), mainly including “721” (Marsh→Dry farmland→Paddy, 27.10%) and “731” ((Marsh→Grassland→Paddy, 12.64%), which reveals that the marshland in the SSP was reclaimed or forested on a large scale during period 1 (1965–1995) and then was transformed into paddies during period 2 (1995–2015). Marsh reclamation for cultivated land still accounted for a relatively large proportion of two-steps trajectories during this period. The two-step changed trajectories also represent the conversion from marsh to grassland, as well as that between marsh and water, which may have a relationship with changes in rainfall.

The spatial patterns of unchanged, naturally induced, and human-induced changes are shown in Figure 5b. Statistics indicate that the proportion of human-induced types is the biggest (79.95%), while those of unchanged and naturally induced types only constitute 7.60% and 12.44%, respectively. Additionally, the proportion of natural evolution leading to marsh loss was much larger than natural evolution leading to marsh gain. Thus, human activities have played a major role in marsh loss since 1965 in the SSP.

## 4. Discussion

### 4.1. Uncertainty Analysis

Integrating multi-source RS images is the most economically feasible way to acquire historical land-use maps in many cases. However, combining multi-source RS images may produce some limitations, such as different spectral and spatial resolutions. In order to solve the limitation of inconsistent spatial resolution, CORONA images were resampled to 30 m in this study, which may lead to the loss of some information. Considering the limitation of different spectral resolutions, visual interpretation, which can combine multi-source information such as textures and shapes, was used in this study. However, visual interpretation is time consuming and laborious. Therefore, under the premise of ensuring accuracy, classifying multi-source information in combination with automatic classification to improve efficiency needs to be strengthened in the future. The lack of related historical land-use data creates a problem of accuracy validation [47,48]. To ensure the accuracy of land-use data in this study, extensive field surveys were performed in the 1990s and 2015. Photos taken by a camera during field surveys were used to validate land-use data in this study. The verification points were randomly chosen at a ratio of 10%. Historical records, such as aerial images and statistical data, and interviews with local residents and experts were also used to validate the accuracy. The land-use data in 1965 in this study was compared with a field survey of marshes from the 1960s by the Northeast Institute of Geography and Agroecology, the Chinese Academy of Sciences. UAV images from 2015 were used for accuracy verification and as a supplement for the classification of land-use. In this study, UAV images, which can provide more detailed information, were used as a supplement to validate our land-use map from 2015. Because marshes are always located in areas with poor traffic, UAVs can provide great convenience in the field surveys of marshes. For example, a UAV can detect marshes blocked by forests. The overall accuracy of the four classes (forest land, grassland, water body, and settlement) was no less than 94.3%, and that of the four subclasses (paddy field, dry farmland, marsh, and other unused land) was no less than 91.2%. There are still some uncertainties in this study. Due to time and economic constraints, this study does not compare the land-use extraction results obtained from CORONA and Landsat images captured at a similar time (e.g., the 1970s). To maintain data consistency, the CORONA images were resampled to 30 m in this study, which may have reduced the accuracy to some extent. In general, the land-use maps in this study are credible, despite some uncertainties.

To distinguish the role of human activities and natural factors, we assumed that marsh changes are human-induced types, provided that one of the observed land-use changes is human-induced, whenever it occurs. Our hypothesis may have some uncertainties to some extent, but it provides a preliminary estimation of the effects of human disturbance on marsh loss. Firstly, once a marsh is destroyed by human beings, it tends to stay stable. For example, once a marsh is converted to cultivated land and settlement, it usually maintains a stable status [24]. Secondly, the proportion of marsh changes caused by human disturbances in period 1 and those induced by natural evolution in period 2 was small. As a result, the hypothesis in this study is relatively reasonable.

### 4.2. The Role of Human Activities

This study clarifies the dominant role of human disturbances quantitatively through a trajectory analysis. Previous studies have also indicated that land-use changes are largely related to human disturbances [47,48,49], especially to population expansion and large-scale agricultural reclamation. Trajectory analysis clarifies that the reclamation of cultivated land is the main driving force of marsh loss and degradation in the SSP. Rapid population growth promoted the intensive reclamation of marshes [47,48]. Previous studies have also attempted to quantitatively clarify the effects of climate change. The study by Zhang et al. [60] revealed that climate factors contributed to 17–30% of marsh loss during 1954–2005 in the Sanjiang Plain. The research of Xue et al. [61] indicated that the effect of climate change was approximately 4.33–5.21% from 1981 to 2010. Our results indicate that human activities contributed to 79.95% of the marsh loss from 1965 to 2015 in the SSP, which was the hinterland of the Sanjiang Plain. The main conclusions are consistent with the above two studies.

Government policy was also an important driving force that affected human activities, as well as marsh changes, in the SSP. The “Great Leap Forward” movement in the 1950s [47] and the “Going to the Countryside and Settling in the Communes” policy published at the beginning of 1970s [18] encouraged people to participate in agricultural activities. Under the influence of these policies, approximately 531.5 thousand people (approximately 81,500 veterans and 450,000 educated youth) took part in the wetland reclamation of cultivated land [47], leading to the conversion from marshes to paddy field and dry farmland (Marsh→Paddy and Marsh→Dry farmland) listed in Table 3. The “Agricultural Modernization” [48] policy published in 1978 introduced modern agricultural machinery and then promoted marsh reclamation extensively. The “Three North Shelter Forest Project” implemented in 1979 promoted the transformation from marsh to forest. The time interval in this study is relatively long. The marsh-to-forest conversion mainly involves the transformation from marsh-to-arable land to forest. Additionally, some government polices have supported the area growth of marshland in the SSP, especially during period 2, when natural reservations received more attention. The wetland restoration project, since the late 1990s, and the establishment of natural reserves, since the late 1980s, have promoted wetland protection [48,49].

### 4.3. The Role of Climate Change

**Figure 6:**
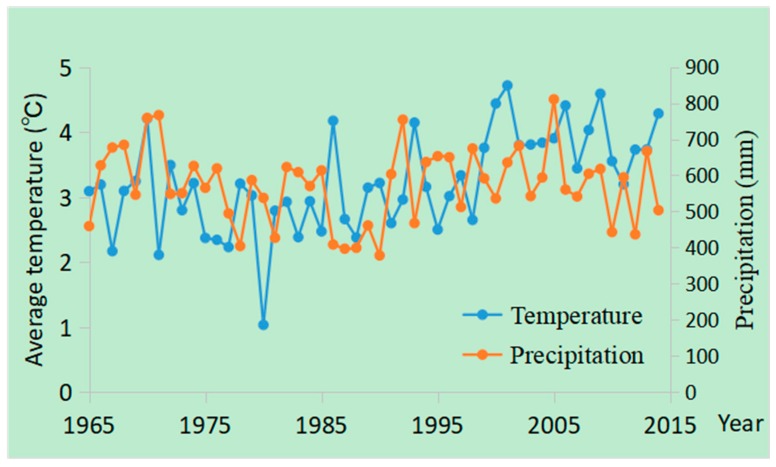
The temperature and precipitation changes since 1965 in the Sanjiang Plain.

Climate change was also an important factor that affected marsh changes in the study area. Climate warming has been observed since the 1950s in Northeast China, as well as in the Sanjiang Plain [18,47,48,49]. Studies indicate that the mean temperature has increased by approximately 0.78 °C per year in the Sanjiang Plain since 1954 [18]. The temperature and precipitation changes since 1965 in the Sanjiang Plain were obtained according to published climate data [18], which were generated by the Kriging interpolation of data from meteorological stations (Figure 6). The results indicate that the yearly average temperature increased by 0.28 °C/10 y, while precipitation decreased at a rate of 0.24 mm/year in the Sanjiang Plain in the past five decades.

The increasing temperature has helped reclamation from marshland in the SSP, given the fact that temperature is one of the most critical environmental variables that affects crop growth, especially in cold regions [62,63]. For example, previous studies indicate that rice cannot be planted in areas where the yearly mean temperature is low [64,65]. Therefore, climate warming is favorable for the conversion from marshes to paddy fields (Marsh→Paddy), as well as for the changes from marshland to dry farmland (Marsh→Dry farmland) listed in Table 3. Despite the fact that the yearly mean precipitation indicated no obvious trends since the 1950s, drought frequency has increased in the Sanjiang Plain, promoting marsh loss in the study area [64]. As an important part of marsh wetlands, the water supply can greatly influence marsh distribution, as well as the conversion between marsh and grassland/water [66,67,68]. Increased drought frequency promotes the transformation from marsh to grassland/water listed in Table 3. Wind speed has obviously declined in the past several decades in Northeast China [48], which has been beneficial for crop growth. Decreased wind speed promoted the transformation from marsh to paddy and from marsh to dry farmland, as listed in Table 3. Both climate warming and decreased wind speed promoted the transformation from marsh to cultivated land.

### 4.4. Wetland Conservation and Restoration

The marsh loss rate has declined in the past five decades in the SSP. One important reason for this decline is that wetland protection is valued. However, it cannot be ignored that the total area of marshland still shows a downward trend. Therefore, wetland conservation and restoration still need to be taken seriously. Wetland conservation can be strengthened by building more wetland nature reserves, strengthening environmental assessments of wetland development projects, and enhancing public awareness of wetland protection [69,70,71]. Wetland conservation can reduce the loss of unchanged types (Marsh→Marsh) listed in Table 3. Restoring water in a wetland environment has become the key to solving wetland restoration problems [72,73,74]. Water restoration helps convert grassland and other unused land to wetlands (Grassland→Marsh and Other unused land→Marsh listed in Table 3). Human disturbances, such as road construction and trench digging, have changed the original landform features of this land, which has led to wetland hydrological disconnection [75]. Therefore, before the restoration of water depth, these patches need to be connected naturally through micro-landscape transformation to ensure that water depth gradients are diverse, as well as the normal growth of vegetation under different water depth conditions during wetland restoration [74,76]. Considering that reclamation is the main cause of wetland loss, promoting the conversion of cultivated land to wetland is a priority for wetland restoration [77,78]. The transformation from paddy to marsh (Paddy→Marsh) and from dry farmland to marsh (Dry farmland→Marsh), as listed in Table 3, can be implemented to promote wetland restoration. Apart from the ecological aspects of protecting and restoring wetlands, the economic benefits of wetlands also cannot be ignored [69,71]. Some actions can be taken to promote the rational use of wetland resources in the SSP. The introduction of aquatic economic plants, such as lotus seed, can be carried out to increase biodiversity and economic benefits [79,80,81]. Fish and crab can be raised in wetland areas to increase economic income and biodiversity [82,83]. Reeds can be used to raise crabs and achieve a reed–crab compound ecosystem.

## 5. Conclusions

Monitoring historical wetland dynamics and related land-use changes is essential to understand the wetland evolution process. Quantitatively assessing the impact of human activities on wetland changes can help support knowledge-based conservation policies. Combining multi-source RS images, this study identified marsh dynamics and driving factors in the SSP from 1965 to 2015. The results indicate that the marsh area in the SSP decreased dramatically during 1965–2015, with a drastic drop of 53.17% during the study period. The marshland was mostly transformed into cultivated land in the SSP from 1965 to 2015, indicating that agricultural encroachment was the dominant contributor to marsh degradation in the SSP. The analysis of landscape indexes indicates that marsh fragmentation has been aggravated over the past five decades. Trajectory analysis also shows that human activities have acted as the primary driving force behind marsh changes in the SSP since 1965.

## Figures and Tables

**Table 1:** The landscape metrics.

Type	Unit	Description
AREA_MN	ha	Mean area stands for the mean patch size.
LSI	None	Landscape shape index that provides a standardized measure of the total edge or edge density and is only meaningful relative to the size of the landscape.
NP	None	NP means the number of patches of a given landscape type.
DIVISION	None	Landscape division index equals 1 minus the sum of the patch area (m^2^) divided by the total landscape area (m^2^). A greater DIVISION represents more fragmentation.
SPLIT	None	Splitting index equals the total landscape area (m^2^) squared divided by the sum of the patch area (m^2^) squared. Greater SPLIT means more fragmentation.
AI	%	Aggregation index equals the number of like adjacencies involving the corresponding class. The smaller the AI index means less aggregation.

**Table 2:** Land-use change types.

Type	Examples of Trajectory Code
Paddy field	“122” indicates that the parcel transformed from paddy to dry farmland from 1965 to 1995 and then kept as dry farmland during 1995–2015.
Dry farmland	“721”indicates that the parcel transformed from marsh to dry farmland from 1965 to 1995 and then converted to paddy during 1995–2015.
Forest land	“321”indicates that the parcel transformed from forest land to dry farmland during 1965–1995 and then converted to dry farmland from 1995 to 2015.
Grassland	“742”indicates that the parcel transformed from marsh to grassland during 1965–1995 and then converted to dry farmland from 1995 to 2015.
Water body	“588”indicates that the parcel transformed from water body to unused land from 1965 to 1995 and then kept as unused land during 1995–2015.
Settlement	“726”indicates that the parcel transformed from marsh to dry farmland from 1965 to 1995 and then converted to settlement during 1995–2015.
Marsh	“711”indicates that the parcel transformed from marsh to paddy from 1965 to 1995 and then kept as paddy during 1995–2015.
Other unused land	“788”indicates that the parcel transformed from marsh to unused land from 1965 to 1995 and then kept as unused land during 1995–2015.

**Table 3:** Land-use change types related to marsh changes.

Type	Land-Use Changes	Examples
Human-induced type	Marsh→Paddy	711; 771; 721
	Marsh→Dry farmland	722; 772; 712
	Marsh→Forest	733; 773; 732
	Marsh→Settlement	766; 776; 726
Natural evolution leading to marsh gain	Paddy→Marsh	177; 117
	Dry farmland→Marsh	277; 227
	Forest→Marsh	377; 337
	Grassland→Marsh	477; 447
	Water→Marsh	577; 557; 575
	Other unused land→Marsh	877; 887; 878
Natural evolution leading to marsh loss	Marsh→Grassland	744; 774; 747
	Marsh→Water	755; 775; 757
	Marsh→Other unused land	788; 778; 787
Unchanged type	Marsh→Marsh	777

**Table 4:** Marsh loss rate in different time intervals.

Period	Loss Area (ha)	Annual Loss Area (ha/year)	Loss Rate (%)
1965–1995	−719,486.55	−23,982.89	−68.96
1995–2015	−133,047.16	−6652.36	−41.08

**Table 5:** Landscape changes of the marsh in the SSP in the past five decades.

Year	NP	LSI	AREA_MN	DIVISION	SPLIT	AI
1965	1383	49.7857	754.7203	0.6759	3.085	98.5668
1995	662	41.3445	489.2211	0.9948	194.014	97.8721
2015	489	32.1449	390.1984	0.9986	696.4322	97.8589

## Supplementary Materials

The following are available online at https://www.mdpi.com/1424-8220/20/4/1036/s1, Table S1: Percentages (>0.01%) of one-step changes by codes, 1965–2015 (%), Table S2: Percentages (>0.01%) of two-steps changes by codes, 1965–2015 (%).
