# Supplementary material for: Large-Scale Marsh Loss Reconstructed from Satellite Data in the Small Sanjiang Plain since 1965: Process, Pattern and Driving Force
Source: Sensors (Basel). 2020 Feb 14;20(4):1036. doi: 10.3390/s20041036 (PMC7070650; doi:10.3390/s20041036)
Supplement: Supplementary file 1 [file sensors-20-01036-s001.pdf]

Table S1. Percentages (>0.01%) of one-step changes by codes, 1965–2015 (%).

Code	%	Code	%	Code	%	Code	%
771	10.89	711	1.55	447	0.20	755	0.02
772	5.31	774	1.26	766	0.11	744	0.01
733	2.55	788	0.86	477	0.09	776	0.01
778	2.39	337	0.29	557	0.03	775	0.01
722	2.35	773	0.20	377	0.03	577	0.01

Table S2. Percentages (>0.01%) of two-steps changes by codes, 1965–2015 (%).

Code	%	Code	%	Code	%	Code	%	Code	%
721	27.10	757	0.39	373	0.11	713	0.03	374	0.01
731	12.64	723	0.35	742	0.10	372	0.03	717	0.01
738	4.81	747	0.33	371	0.07	784	0.03	237	0.01
787	3.70	472	0.31	427	0.07	473	0.03	537	0.01
737	3.60	782	0.21	378	0.06	327	0.02	753	0.01
728	2.43	748	0.19	761	0.05	785	0.02	752	0.01
732	2.01	575	0.15	736	0.04	751	0.02	725	0.01
471	1.17	726	0.14	781	0.04	587	0.02	783	0.01
734	1.05	474	0.13	478	0.04	762	0.02	417	0.01
724	0.86	741	0.12	714	0.04	271	0.02	764	0.01
727	0.71	758	0.12	743	0.04	735	0.02	272	0.01
437	0.51	712	0.11	578	0.04	716	0.01	374	0.01
